# Lactobacillus crispatus L1: high cell density cultivation and exopolysaccharide structure characterization to highlight potentially beneficial effects against vaginal pathogens

**DOI:** 10.1186/1471-2180-14-137

**Published:** 2014-05-30

**Authors:** Giovanna Donnarumma, Antonio Molinaro, Donatella Cimini, Cristina De Castro, Vivien Valli, Vincenza De Gregorio, Mario De Rosa, Chiara Schiraldi

**Affiliations:** 1 Department of Experimental Medicine, Section of Biotechnology and Molecular Biology, Second University of Naples, via De Crecchio n°7, Naples 80138, Italy; 2 Department of Experimental Medicine, Section of Microbiology and Clinical Microbiology, Second University of Naples, via De Crecchio n°7, Naples 80138, Italy; 3 Department of Chemical Sciences, University of Naples Federico II, via Cinthia 4, Naples 80126, Italy

**Keywords:** *L. crispatus* L1, High cell densities cultivation, Simulated digestion

## Abstract

**Background:**

Vaginal lactic acid bacteria defend the host against pathogens through a combination of competitive exclusion, competition for nutrients, production of antimicrobial substances and through the activation of the immune system. A new human isolate named *Lactobacillus crispatus L1* was characterized in this work*,* and a preliminary evaluation of its probiotic potential is described together with a process to obtain a high productivity of viable biomass.

**Results:**

In a simulated digestion process 1.8⋅10^10^ cells∙ml^−1^ survived the gastric environment with 80% viability, without being affected by small intestine juices. Experiments on six different C sources were performed to analyze growth and organic acids production and, glucose, provided the best performances. A microfiltration strategy was exploited to improve the cellular yield in 2 L-fermentation processes, reaching 27 g · l^−1^ of dry biomass. Moreover, *L. crispatus* L1 demonstrated a greater stability to high concentrations of lactic acid, compared to other lactobacilli. The specific *L. crispatus L1* exopolysaccharide was purified from the fermentation broth and characterized by NMR showing structural features and similarity to exopolysaccharides produced by pathogenic strains. Live *L. crispatus L1* cells strongly reduced adhesion of a yeast pathogenic strain, *Candida albicans* in particular, in adherence assays. Interestingly a higher expression of the human defensin HBD-2 was also observed in vaginal cells treated with the purified exopolysaccharide, indicating a possible correlation with *C. albicans* growth inhibition.

**Conclusions:**

The paper describes the evaluation of *L. crispatus* L1 as potential vaginal probiotic and the fermentation processes to obtain high concentrations of viable cells.

## Background

Lactobacilli have long been of interest to the dairy and agriculture industries, in fact, they are defined as *generally regarded as safe* (e.g. through regulatory agency), and some have been found as ubiquitous members of the mucosae of healthy subjects [1]. Some studies describe the use of lactic acid bacteria (LAB) for the treatment or prevention of infections of the intestinal and genital tracts with different extents of success [2,3]. It is quite difficult to identify which properties of lactobacilli are required to prevent and eventually treat diseases and to determine the adequate dosage, duration, and methods of delivery.

In respect to vaginal probiotics, the protective role of lactobacilli seems to be based upon two mechanisms, namely, the specific adherence to the vaginal epithelium leading to intensive colonization of this surface, and the control of the remaining vaginal microflora through antagonism against pathogens. As a consequence, the ability of *lactobacillus* to adhere to epithelial cells and mucosal surfaces is a key criterion for the selection of probiotics [4].

The efficacy of the available commercial products is also strictly dependent on the viability of the probiotic strains contained in the preparations, since the amount of applied microorganism could be crucial for the effectiveness of the product [5], and several studies revealed that some health food products did not satisfy the claims stated on the labels therefore minimizing the expected health benefits [6]. Therefore the evaluation of cell viability in conditions that mimic the practical application is a key issue in the selection of probiotics.

Also the development of novel fermentation strategies to increase the final biomass yield is central to bypass one of the bottlenecks encountered in the production of starters, probiotic ingredients and medical devices. However, since their growth is inhibited by their primary metabolic product (pH lowering but also lactate effect in buffered cultivations), lactobacilli are rarely cultivated at high cellular density (i.e. higher than 8⋅10^9^ cells∙ml^−1^) in batch/fed-batch fermentation processes.

Vaginal probiotics are a rather new area of investigation and, therefore, not much is known about the mechanisms, the conditions or characteristics needed to assess their efficacy. Several strains appear to be effective in colonizing and then protecting the intestine and the urogenital tract [7-9], from infections. Commercial lactobacilli-based products such as Normogin® have demonstrated to be a reliable treatment for reducing the recurrence of bacterial vaginosis [10]. It has been reported that infection mechanisms are mainly due to a disestablishment of the normal resident vaginal microflora, primarily a loss of H_2_O_2_-producing lactobacilli [11,12], although some studies do not support this hypothesis [13]. *In vitro* studies have suggested that the re-colonization of the urinary tract by certain specific strains of lactobacilli seems to be a suitable approach to prevent infections and relapses [14,15]. Recently it has also been suggested that some probiotic bacteria could be effective not only when locally delivered (e.g. vaginal instillation) but also when assumed *per os*[16], and this establishes a link between the rate of intestinal survival and vaginal colonization [17].

*Lactobacillus crispatus* can persist in the gastrointestinal tract [18] and is among the most prevalent species of the Lactobacillus-dominated human vaginal microbiota [19], and resistance to very low pH conditions have also been described [20].

A strain of *L. crispatus* (named *L. crispatus* L1) isolated from the vaginal flora of a healthy woman was characterized in this study. In particular, the ability of *L. crispatus* L1 to survive to an *in vitro* simulated digestion was evaluated and its physiological and metabolic requirements were investigated. Optimal growth conditions were defined, in order to obtain high density cultivations needed for potential applications of this strain as probiotic supplement. The use of an *in situ* product removal fermentation process allowed a 7-fold improvement of the biomass yield compared to traditional processes, accompanied by an extremely high cellular viability (94%). Given the necessity of probiotic preparations to deliver a certain amount of viable microbial cells the effect of different protective agents on freeze-drying procedures was also investigated.

Moreover, in order to investigate on the chemical nature of the agents that are at the basis of the beneficial effect of *L. crispatus* L1 we have established the primary structure of its exopolysaccharides (EPS), since previous studies [21,22] on bacterial adhesion showed that EPS might promote the adherence of bacteria to biological surfaces, thereby facilitating the colonization of various ecological niches. Intriguingly, the EPS resulted to be a mannan polysaccharide possessing a structure very similar to the one produced by *Candida albicans*[23]. The EPS was found to be present in adherent biofilms and might be involved in initial and permanent adhesion. Moreover the EPS-induced increased expression of the human defensin HBD-2 in vaginal cells was also verified, identifying a possible connection with *C. albicans* growth inhibition [24].

## Results

### Strain identification and H_2_O_2_ production

A *Lactobacillus* strain isolated from human vaginal secretion was allotted to *crispatus* subspecies by 16S ribosomal DNA sequencing [25] and it was named *L. crispatus* L1. In particular, PCR products were pooled, purified and sequenced.

In addition, the ability of 72 *Lactobacillus* strains to produce H_2_O_2_ was evaluated. The percentage of strains classified as strong, medium, weak and negative H_2_O_2_ producers was 23, 34, 38 and 5%, respectively. *L. crispatus* L1 was found to be the best of the isolates in the laboratory collection.

### *In vitro* digestion

**Figure 1:**
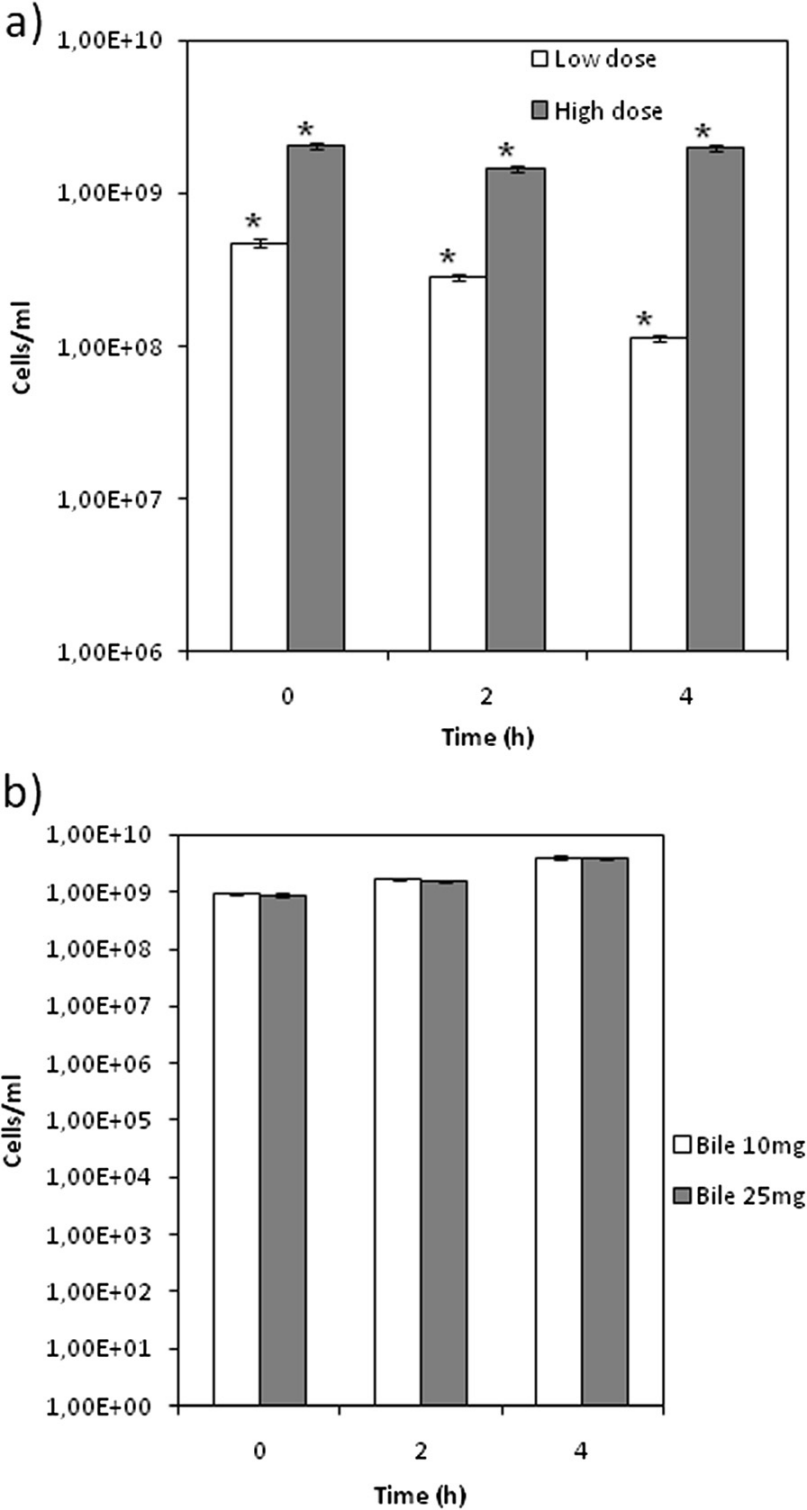
**Simulation of human digestion in shake flasks. (a)** Survival of *L. crispatus* L1 to gastric juices (pH 2.0, pepsine 3 g∙l^−1^). Response of different doses of bacteria, high (1.8 · 10^9^ cells∙ml^−1^) and low (6.0 · 10^8^ cells∙ml^−1^), to the treatment. **(b)** Survival of *L. crispatus* L1 to pancreatic juices (pH 4.0, pancreatine 2 g∙l^−1^, Oxgall in different concentrations). Effect of two different concentrations of bile salts on the viability of 1.0 · 10^9^ cells∙ml^−1^.The asterisks indicate a statistically significant difference between samples with P < 0.01.

Results from shake flask experiments simulating the passage through the gastrointestinal tract showed a good resistance of *L. crispatus* L1 to the *in vitro* digestion process. The bacterial dose significantly influenced results, as shown in Figure 1a clearly indicating that 1.8⋅10^9^ cells∙ml^−1^ corresponds to the minimal required initial concentration of cells necessary to survive gastric juices. Incubation in simulated pancreatic juices (Figure 1b) with different Oxgall concentrations (10 mg and 25 mg) did not affect viability, whereas a slight increase of the cell number within 4 h was observed. Moreover, treated cells reached a final biomass yield comparable with that of the control cells (data not shown).

### Shakeflask experiments

A semidefined medium containing soy peptone (10 g∙l^−1^) and yeast extract (2.5 g∙l^−1^) was used to investigate the amount of biomass and lactic acid produced using different carbon sources (Table 1). The final titer of biomass produced in shake flasks was very similar in all the media analysed. The production of lactic acid was quite high ranging between 7.5 and 13.1 g∙l^−1^ (Table 1) and resulting in relevant Y_p/s_ ranging between 0.68 and 0.89 g∙g^−1^. The Y_p/s_ on dextrins could not be calculated due to the presence of high molecular weight carbohydrates (glucose residues >7) that were not degraded and metabolized as evidenced by High Performance Anion Exchange Chromatography with Pulsed Amperometric Detection (HPAEC-PAD) analyses.

**Table 1:** **Growth of *L. crispatus *** **L1 in shake flasks on SDM medium supplemented with different carbon sources**

**Substrate (20 g · l**^**−1**^**)**	**Cell dry weight (g · l**^**−1**^**)**	**μ**_**8h**_**(h**^**−1**^**)**	**Lactic acid (g · l**^**−1**^**)**	**Y**_**x/s**_**(g · g**^**−1**^**)**	**Y**_**p/s**_**(g · g**^**−1**^**)**
**Glucose**	1.61	0.34	8.82	0.15	0.83
**Sucrose**	1.51	0.46	13.10	0.13	0.68
**Lactose**	1.35	0.24	8.00	0.15	0.89
**Trehalose**	1.50	0.43	9.21	0.12	0.74
**Fructose**	1.51	0.34	7.50	0.18	0.78
**Dextrins**	1.61	0.31	11.0	n.d.	n.d.

The concentration of biomass and lactic acid were measured in the broth after 24 h of growth. Y_x/s_ indicates g of dry biomass produced per g of substrate; Y_p/s_ indicates g of lactic acid produced per g of substrate; μ8h indicates the specific growth rate in h^−1^ calculated in the first 8 h of growth. Values are an average of 3 different experiments with standard deviations ≤ 5%.

### Batch and microfiltration fermentation processes

Glucose and sucrose were selected as carbon sources for the following batch experiments.

**Figure 2:**
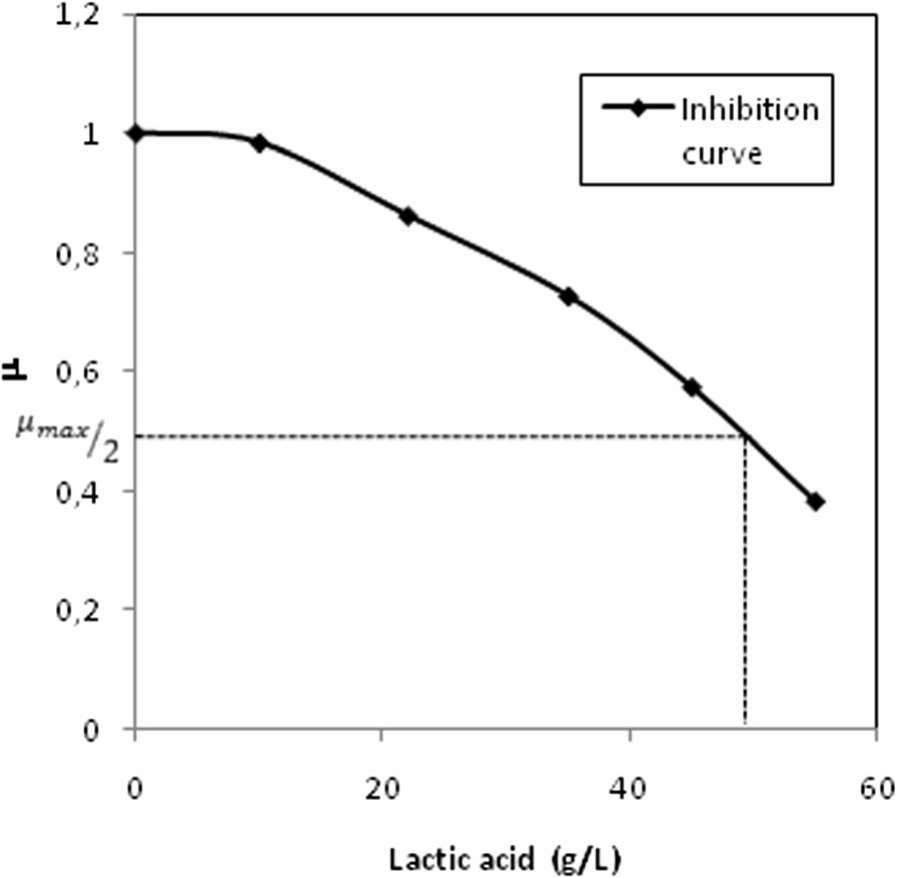
**Lactate inhibition curve.** The graph shows the specific growth rate of *L. crispatus* L1 using increasing concentrations of sodium lactate in the medium at pH 6.5.

**Figure 3:**
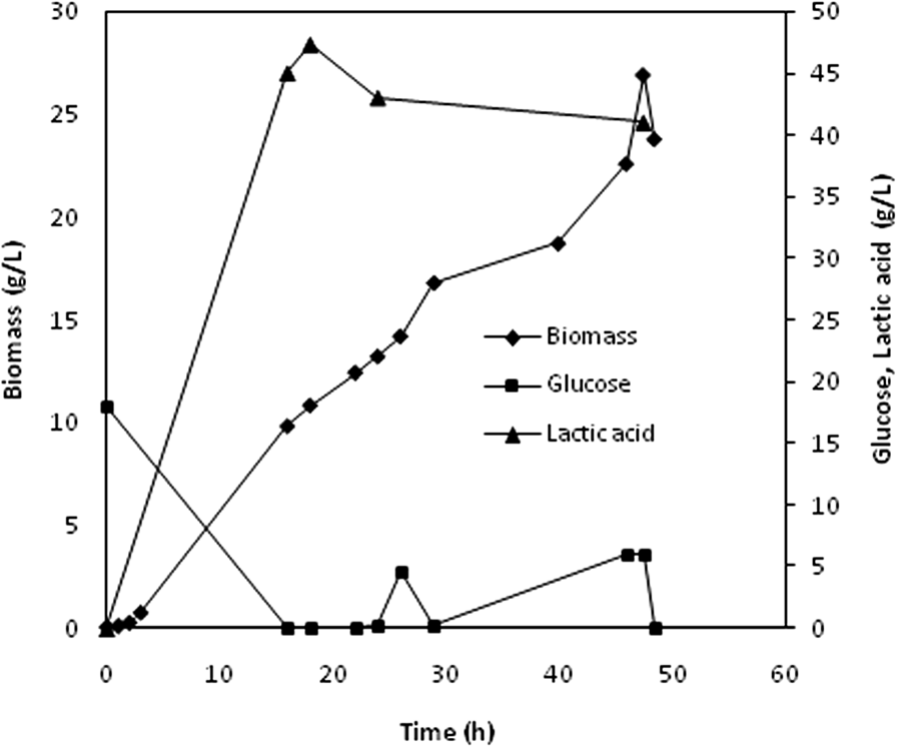
**Growth of *L. crispatus *L1 in a microfiltration experiment.** Time course of biomass, production of lactic acid and residual glucose on SDM.

During these experiments *L. crispatus* L1 demonstrated a similar growth rate and final concentration of cells. The maximum titer of biomass on the two substrates was slightly different, in particular, 3.9 ± 0.2 g_cdw_∙l^−1^ were obtained on glucose and 3.4 ± 0.1 g_cdw_∙l^−1^ on sucrose (Table 2). The final amount of lactic acid was also quite similar, and it corresponded to 12 and 14 g∙l^−1^ on glucose and sucrose, respectively. Product (lactate) inhibition was also studied to better characterize the physiology of *L. crispatus* L1. Increasing amounts of sodium lactate added to the SDM medium at a fixed pH lowered the initial specific growth rate (1–3 h). In particular, μ appeared to be reduced by half with 45 g∙l^−1^ lactate (Figure 2). In order to dilute lactic acid and overcome inhibition problems, a bioreactor with microfiltration modules was used to perform *in situ* product removal experiments (Figure 3). A maximum of 27.1 g_cdw_∙l^−1^ in 45 h of growth were produced with a final concentration of 46 g∙l^−1^ of lactic acid. As it is shown in Table 3, a 7-fold improvement of the final titer of biomass was achieved by microfiltration experiments compared to previous batch processes. Moreover the total amount of lactic acid produced was equal to 148 g (ϕ = 0.37 g∙l^−1^∙h^−1^) with a Y_p/s_ of 0.75 g∙g^−1^ (Table 3). All results presented are average of at least 3 experiments.

**Table 2:** **Yield of biomass and lactic acid obtained in batch experiments of *L. crispatus *** **L1 grown on SDM supplemented with 20 g · l**^**−1 **^**glucose or sucrose as main carbon sources**

**Carbon source**	**Cell dry weight (g · l**^**−1**^**)**	**Lactic acid (g · l**^**−1**^**)**	**μ**_**max**_**(h**^**−1**^**)**
**Glucose**	3.8 ± 0.3	11.5 ± 0.5	0.84
**Sucrose**	3.3 ± 0.2	13.6 ± 0.4	0.60

The medium contained soy peptone and yeast extract as nitrogen sources.

**Table 3:** **Comparison of yields and productivities obtained in batch and microfiltration experiments of *L. crispatus *** **L1**

	**Cell dry weight (g · l**^**−1**^**)**	**Lactic acid (g · l**^**−1**^**)**	**Biomass productivity (g · l**^**−1**^ **· h**^**−1**^**)**	**Lactic acid productivity (g · l**^**−1**^ **· h**^**−1**^**)**	**Y**_**x/s**_**(g · g**^**−1**^**)**	**Y**_**p/s**_**(g · g**^**−1**^**)**
**Batch**	3.8 ± 0.3	11.5 ± 0.5	0.20 ± 0.07	0.40 ± 0.02	0.20 ± 0.02	0.57 ± 0.03
**MF**	27.31 ± 1.5	46.02 ± 2.3	0.60 ± 0.03	0.70 ± 0.07	0.13 ± 0.08	0.75 ± 0.04

Y_x/s_ indicates g of dry biomass produced per g of substrate; Y_p/s_ indicates g of lactic acid produced per g of substrate. Values are an average of 3 different experiments.

### EPS production and purification

The EPSs content in the fermentation broth ranged between 200–400 mg⋅l^−1^ and the initial protein titre was estimated up to 50 fold. Protease was used to eliminate these major contaminants of the exopolysaccarides, and the following tangential ultrafiltration (UF)/ diafiltration (DF) was performed to further purify the product and to remove salt and other smaller contaminants. During UF the flux decreased from 8.3 to 7.3 l∙m^−2^ h^−1^ and it increased again to 13.5 l∙m^−2^ h^−1^ during the DF phase that lasted until reaching a conductivity of 0.8mS/cm. The supernatant was concentrated 9 fold compared to the initial volume.

The recovery yield after membrane purification was on average 85% and the purified EPSs solution had a protein content that was inferior to 0.5% w/w.

### Structure determination of mannan polymer

**Figure 4:**
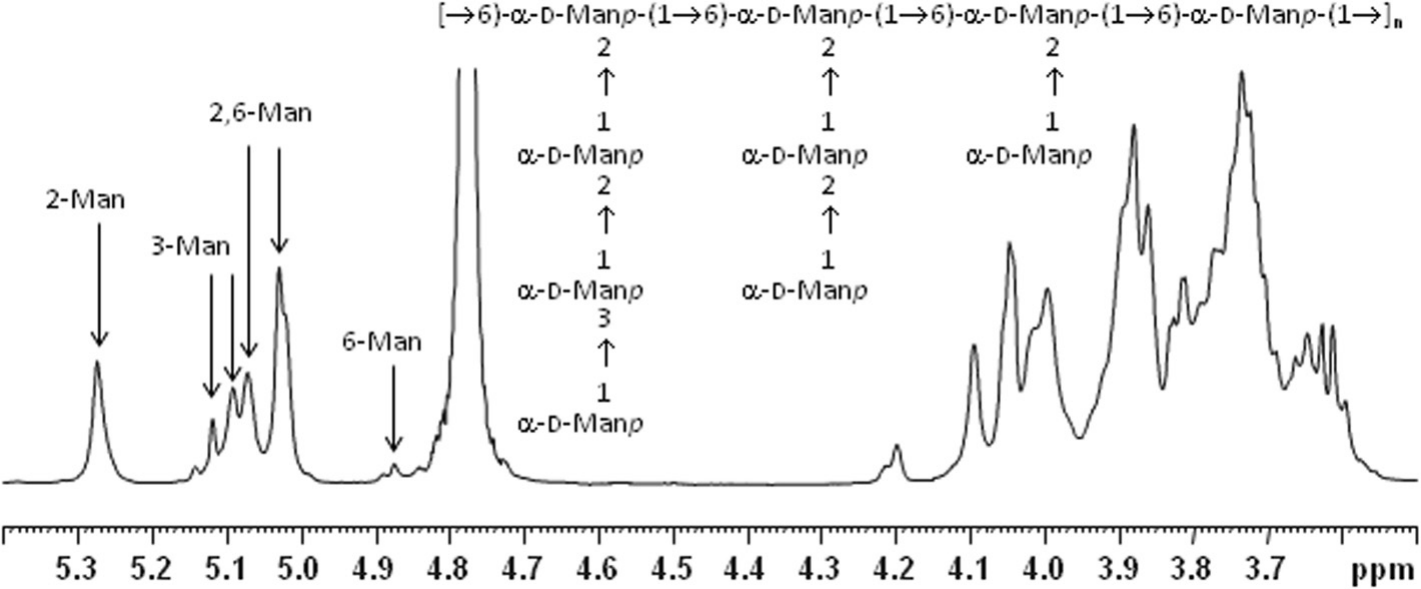
**Characterization of the EPS produced by *L. crispatus *L1.**^1^H-NMR spectrum and spin system attribution for each sugar of the mannan polysaccharide and structure of the EPS.

Compositional and methylation analyses showed the presence of different derivatives of mannose, such as terminal Man*p*, 2-substituted Man*p*, 3-substituted Man*p*, 6-substituted Man*p* and 2,6-substituted Man*p*. On this ground, it could be deemed the presence of a very intricate polymer only based on a mannose monosaccharide, in which other mannose branching residues were attached to a mannan backbone. The polysaccharide underwent Nuclear Magnetic Resonance (NMR) analysis and even though the ^1^H- (Figure 4) and ^13^C-NMR spectra appeared rather complex, it was clearly related to the mannan polysaccharides already described [26]. 2D NMR and degradation procedures confirmed the structure, a 6-substituted mannan backbone with small branching chains (one to three units) of Man*p* residues (Figure 4).

### Inhibition of *C. albicans* adhesion to Vk2/E6E7

*C. albicans* is a constituent of the vaginal microbiota and, as opportunistic pathogen, it causes genital infections in humans. In immuno-compromised individuals, overgrowth of the fungus results in candidiasis*. C. albicans* pathogenecity depends on several virulence traits that allow the fungus to invade new tissues, evade the immune system of the host, and facilitate the infection [27]. To verify the antagonist effect of *L. crispatus* L1 against *C. albicans*, the influence of the strain on the adhesion capacity of *C. albicans* to immortalized human vaginal epithelial cell line was evaluated.

**Figure 5:**
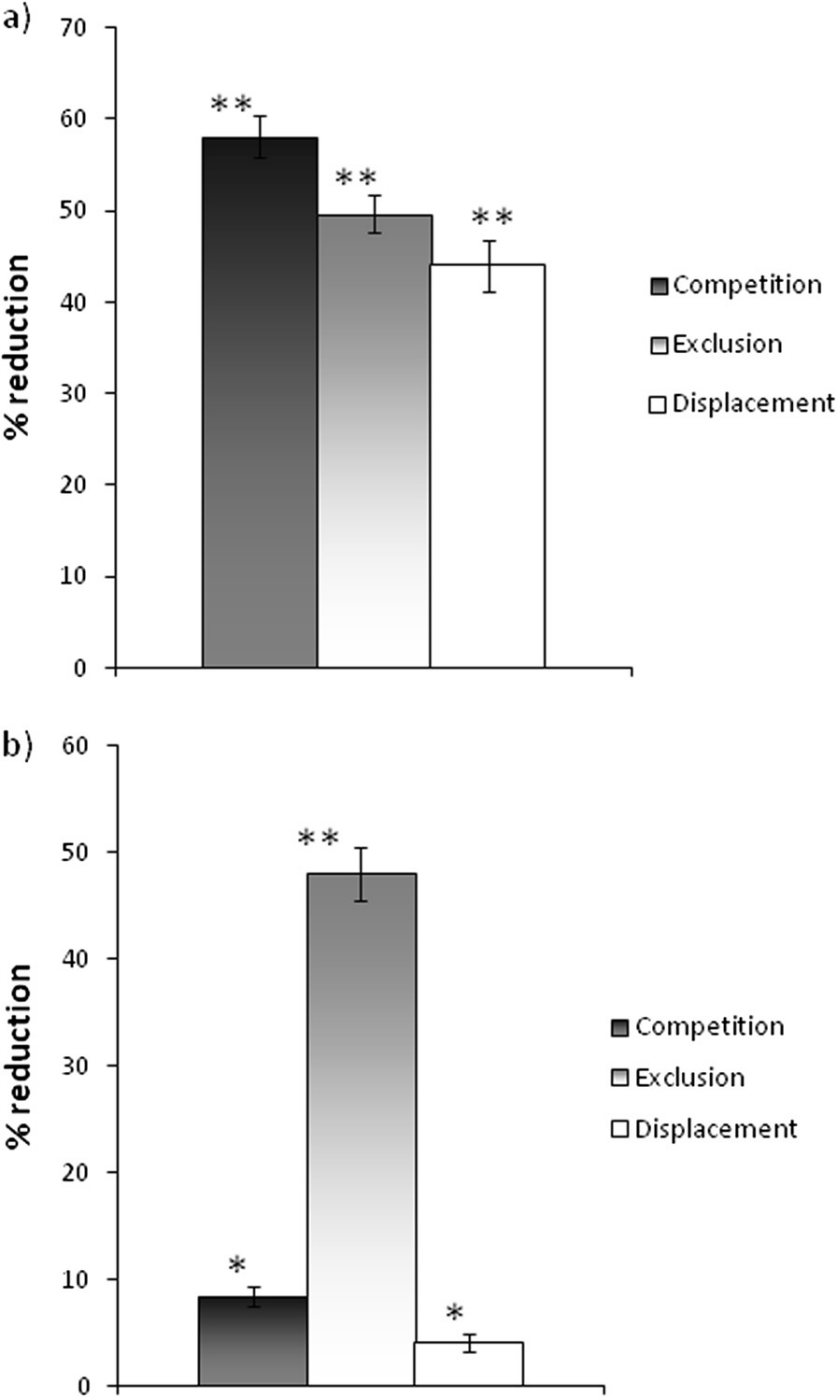
**Inhibition of adhesion of *C.albicans *to vaginal epithelial cells. (a)** Treatment of vaginal epithelial cells with 1×10^7^*L. crispatus. C. albicans* to Vk2/E6E7 cells was assessed by microscopy (×100) after Gram’s stain by counting the number of micro-organisms attached to 30 consecutive cells. The results of the three conditions (i.e. exclusion, competition and displacement) were expressed as the average number of *C. albicans* per Vk2/E6E7 cells and compared with adhesion without lactobacilli (control value). The control values were taken as 100% of adhesion and the inhibition of *C. albicans* adherence was calculated by subtracting each adhesion percentage from their corresponding control value. **(b)** Treatment of vaginal epithelial cells with 1.0 mg/mL EPS. *C. albicans* to Vk2/E6E7 cells was assessed by microscopy (×100) after Gram’s stain by counting the number of micro-organisms attached to 30 consecutive cells. The results of the three conditions (i.e. exclusion, competition and displacement) were expressed as the average number of C. albicans per Vk2/E6E7 cells and compared with adhesion without EPS (control value). The control values were taken as 100% of adhesion and the inhibition of *C. albicans* adherence was calculated by substracting each adhesion percentage from their corresponding control value. The data are expressed as the mean ± SD percentage of adherence in three independent experiments. The asterisks indicate a statistically significant difference between *C. albicans* grown in the presence of viable or heat-killed *L. crispatus* versus *C. albicans* alone. *P < 0.05, **P < 0.01.

The results demonstrate that there is a significantly reduced adhesion of *C. albicans* to Vk2/E6E7 cells by 58 ± 2.4, 49 ± 2.0 and 44 ± 2.8% in the competition, exclusion and displacement assays, respectively (Figure 5).

Moreover, confluent cell monolayers were treated with increasing concentrations of EPS, isolated and purified from *L. crispatus* L1, and successively infected with *C. albicans*. The concentration required to interfere with yeast adhesiveness was equal to 1.0 mg∙ml^−1^.

Figure 5b shows the effect of EPS on the adhesion of *C. albicans* to vaginal epithelial cells under the conditions of exclusion, competition and displacement. The adhesion interference was of about 48% in the exclusion assay, when the monolayers were pre-treated with 1.0 mg∙ml^−1^ EPS for 18 h and before addition of the *C. albicans* suspension. In the competition and displacement tests the reduction in adherence was comparable to that obtained in the control experiment.

**Figure 6:**
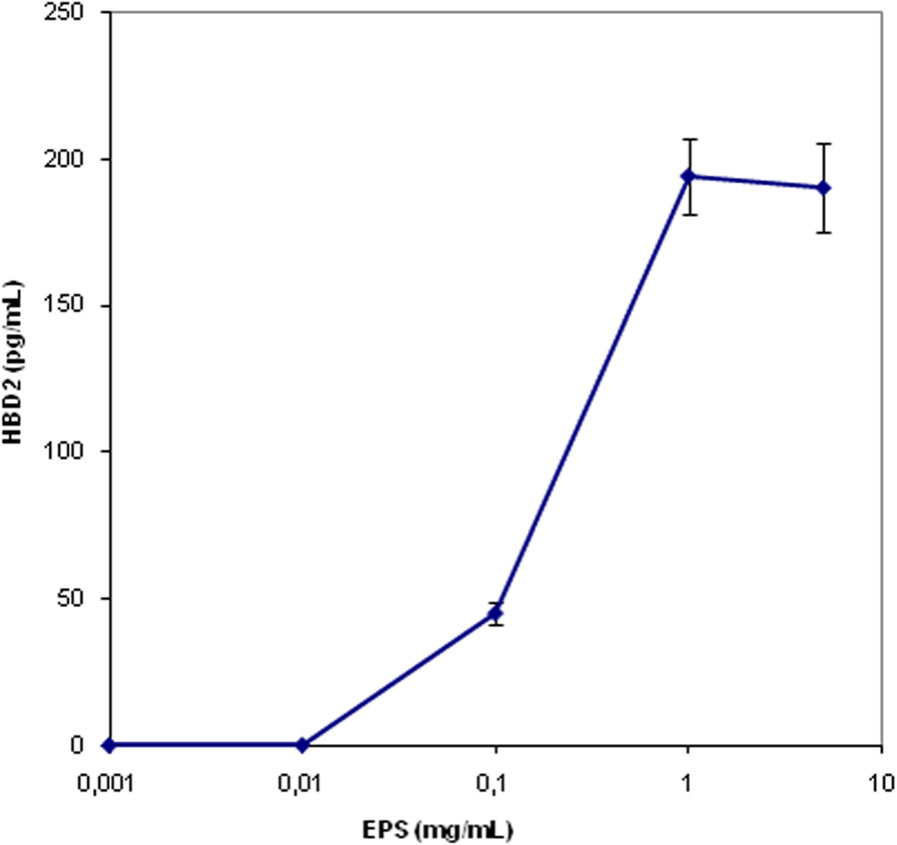
**HBD-2 levels in Vk2/E6E7cells after treatment with EPS (0.01-0.1-1.0 - 5 mg/mL) secreted by *L. crispatus *L1.** The concentration of HBD-2 secreted in supernatant was measured by ELISA.

A set of experiments was performed to determine whether HBD-2 was secreted by vaginal epithelial cells treated with increasing concentrations of EPS. HBD-2 ELISAs showed that the concentration of HBD-2 protein was significantly high in the supernatant after 18 h treatment (Figure 6). Interestingly, the plateau was reached at the same concentration (100 mg∙l^−1^).

## Discussion

Lactobacilli are the prevailing bacteria of the vaginal flora of healthy individuals that regulate the equilibrium between the resident microbiota and the vaginal environment [28]. Cervicovaginal microbiota not dominated by lactobacilli may facilitate transmission of HIV and other sexually transmitted infections. *L. crispatus*, *L. jensenii*, and to a lesser extent *L. gasseri*, are common in the vagina of healthy women, whereas the dominance of *L. iners* is associated with bacterial vaginosis [29]. Borgdorff and colleagues [30] identified six microbiome clusters and concluded that *L. crispatus*-dominated cervicovaginal microbiota are associated with a lower prevalence of sexually transmitted infections and a lower likelihood of genital HIV-1 RNA shedding. Recent literature describes the identification of *L. crispatus* as a member of the resident beneficial flora of the vaginal mucosae [31]. In agreement with this finding the strain isolated in this work from vaginal fluids of a healthy woman was found to belong to this species and named *L. crispatus* L1 .

Vaginal probiotics based on lactic acid bacteria have been proposed as a valid strategy against recurrent infections. LAB use several mechanisms to create an unfriendly environment for pathogens which include the production of antimicrobial substances, such as organic acids, hydrogen peroxide and bacteriocins, and the synthesis of biofilms, in order colonize the vaginal mucosa and displace the infective agents [7,31].

In view of a potential application of *L. crispatus* L1 as vaginal probiotic, it was interesting to characterize the properties of this new isolate due to the capacity of this strain to modify the host microenvironment and therefore possibly deliver health benefits.

The production of lactic acid and hydrogen peroxide were initially investigated and *L. crispatus* L1 demonstrated the ability to produce both metabolites, and compared to other lactobacilli [32] it proved a better resistance to high concentrations of lactic acid, therefore enhancing its competition capacity. Several studies assessed the effectiveness of oral administration of vaginal probiotic bacteria [16,17,33]. For this reason we monitored the resistance of *L. crispatus* L1 to a simulated digestion process by incubating the bacterium in shake flasks at pH 2 in the presence of pepsine. Data showed that strain survival was linked to the dose of treated bacteria, and, that with a starting concentration of 1.8⋅10^9^ cell∙ml^−1^ cell viability was apparently not affected by small intestine juices. *In vitro* assays simulating exposure to pancreatic juices were also performed showing that, unexpectedly, *L. crispatus* L1 was unaffected by the treatment. These data demonstrate the strain’s potential to be orally delivered.

The perspective to use this strain for biotechnological purposes prompted further studies regarding the development of a medium that could enhance strain growth and be suitable for industrial applications. Experiments were initially performed in shake flasks to identify the most suitable carbon source for maximizing the yield of biomass and lactic acid, and sucrose and glucose were chosen for further small scale batch experiments. As shown in Table 2 the growth rate of *L. crispatus* L1 was not affected by the two different carbon sources; a slightly lower Yp/s was obtained with glucose, nevertheless, the latter is often preferred for industrial processes and therefore it was selected for the following fermentation experiments. In order to increase the production of biomass and related product a high cell density fermentation process exploiting a microfiltration strategy was developed to keep the concentration of lactic acid below the toxic threshold for *L .crispatus* L1 (estimated to be 45 g · l^−1^, Figure 3). The feeding strategy avoided the waste of carbon source and determined a 7-fold and a 4-fold increase of the final titer of biomass and lactic acid, respectively, compared to previous batch experiments (Table 3). Based on earlier studies on *L. bulgaricus*[34] a higher improvement of the final biomass concentration was expected. Probably the adhesion of cells to membrane capillaries lowered transmembrane fluxes thus reducing the medium exchange rate. However, the concentration of biomass reached was very high compared to that obtained by cultivating other lactobacilli; moreover, biomass resulted extremely viable (94%) at the end of the experiments (data not shown), valuable result for the foreseen application in medical devices/ food supplements.

Adhesion seems to be one of the key factors determining the colonization of the digestive ecosystem. Consistently the surface characteristics of lactobacilli are expected to contribute in several ways to their interactions with the host gastrointestinal tract and the gut microbiota, affecting their survival, adherence to the host tissue and interactions with themselves and with other bacteria. Since EPS can have important influences on these processes and on the colonization of the host [35,36] we also have investigated the chemical nature of the EPS produced by *L. crispatus* L1. This structure resulted to be a very intricate comb-like mannan polysaccharide that has been already isolated and identified as capsule/EPS/protein bound-EPS in a number of microorganisms, among these in the yeast *C. albicans*[37]. We therefore hypothesised that the similarity of structure between the EPS of *L. crispatus* L1 and the carbohydrate part of mannoproteins and protein bound-polysaccharides excreted by *C. albicans* could be in part responsible for contrasting *C. albicans* infections. For this reason the ability of *L. crispatus* L1 live cells or of the purified EPS to hinder growth of *C. albicans* was analysed by performing adhesion assays with vaginal cells. In particular three mechanisms were evaluated: (ì) exclusion by adhered lactobacilli or EPS, (ii) competition for and (iii) displacement of adherent yeast cells. Live *L. crispatus* cells demonstrated the ability to strongly reduce the adherence of invading yeast cells in all types of assays, the most powerful being the competition modality in which adherence was decreases by 58% compared to the control. The purified EPS only inhibited yeast adhesiveness if pre-incubated with vaginal cells before the addition of *C. albicans*, whereas it was not efficient in competing or displacing yeast cells.

As is known, human defensins, short cysteine-rich cationic proteins, are key components of the innate immune system. The inducible human beta-defensins are antimicrobial peptides with a broad spectrum of antibacterial and antifungal activity. Human beta-defensin 2 (HBD-2) is primarily produced by epithelial cells. The peptide is highly inducible due to various stimuli and has a broad spectrum antimicrobial activity that is cidal for *Candida*.

It was interesting to observe that the pre-treatment of vaginal epithelial cells with EPS induced a high expression of the antimicrobial peptide HBD-2 against *C. albicans*. The up-regulation of HBD-2 might represent a further mechanism of host protection against *Candida* infections. Overall these data indicate that this molecule is at least in part responsible of the impairment of *C. albicans* adhesion to vaginal cells, thus also demonstrating that it has a main role in the beneficial effect of *L. crispatus* L1 as a natural, probiotic, microbicide for vaginal health. In the light of the information above it is not surprising that *L. crispatus* L1 synthesizes a mannan polysaccharide that closely resembles the carbohydrate part of mannoproteins and protein bound-polysaccharides excreted by *C. albicans*. In our opinion, this is a an important step towards the comprehension of the molecular mechanisms at the basis of the probiotic effect of *L. crispatus* ssp.

## Conclusions

The present work describes the identification of a new human isolate named *L. crispatus* L1 and its characterization in order to demonstrate that it meets some of the criteria that identify probiotic strains, such as the ability to produce high titers of lactic acid and H_2_O_2._ In view of its potential application as oral vaginal probiotic, simulated digestion treatments were performed demonstrating its suitability for oral administration.

Growth optimization was initially analysed in shake flasks and following microfiltration experiments allowed reaching high yields of extremely viable biomass, a key prerequisite for probiotic preparations.

The characterization of the structure of the EPS produced by *L. crispatus* L1 showed a similarity with surface molecules produced by *C. albicans* and the inhibition of the adherence of this yeast to vaginal cells in the presence of live *L. crispatus* L1 further suggested an important role of this bacterium as a promoter of vaginal health.

These achievements underlie the potential of *L. crispatus* L1 as orally administered vaginal probiotic, and hence the strain results interesting for biotechnological applications in medical devices and oral supplements.

## Methods

### Materials

Yeast nitrogen base without aminoacids, bacto casitone peptone and soy peptone were purchased from Difco (Becton Dickinson, Le Pont De Claix, France). De Man, Rogosa and Sharpe Medium (MRS), medium M17, bacteriological agar and the AnaeroGen Compact atmosphere generation system for solid state incubation on petri dishes were from Oxoid (Basingstoke, England). All other chemicals used to prepare the semi-defined medium and the buffers were purchased from Sigma-Aldrich (Milan, Italy). A kit containing acetic acid, lactic acid, citric acid, butyrric acid, iso-butyrric acid, succinic acid, oxalic acid, maleic acid was obtained by Supelco (Milan, Italy) for the analytical quantification of organic acids.

### Microorganism and media

Vaginal fluids collected from healthy women (after informed consent) were plated onto lactobacilli selective medium, namely MRS-agar (Oxoid) and incubated in anaerobic conditions (Gas-Pak System; BBL, Becton Dickinson Biosciences) for 48 h at 37°C.

Microorganisms were maintained in MRS-broth as suspended culture (stabs) at −80°C using glycerol (20% w/v) as cryoprotectant. These stabs were used to inoculate pyrex bottles (250 ml) completely filled with culture media to study cell growth and lactic acid production under microaerofilic conditions over a period of 24–30 h at 37°C, in a rotary shaker (HT Aquatron, Infors, Switzerland) at 160 rpm. Experiments were performed by adding different carbon sources (20 g∙l^−1^) to the semi-defined medium, SDM [38]: in particular fructose, sucrose, lactose, trehalose and dextrins were used alternatively to analyze how microbial growth and organic acids production were affected. Shake flask experiments were also performed adding sodium lactate (0–60 g∙l^−1^) at increasing concentrations in the SDM, to evaluate strain growth inhibition.

### Identification methods

Single colonies were collected from MRS plates and characterized with the API 50 CHL system (BioMérieux) according to the manufacturer’s instructions. In order to correctly identify the *Lactobacillus* at species level, 16S ribosomal DNA (rDNA) was sequenced [39]. The sequences of the selected *Lactobacillus*-specific primers LcrisF (AGCGAGCGGAACTAACAGATTTAC) and LcrisR (AGCTGATCATGCGATCTGCTT) confirmed the amplification of a 154-bp fragment of 16S rRNA from the reference strain *L. crispatus* ATCC33820 [40]. Briefly genomic DNA was extracted from pure cultures using a QIAamp DNA mini kit (Qiagen) according to the manufacturer’s instructions. 4 μl of DNA (≈40 ng), in 50 μl reaction mixtures containing 1× Fast Start High Fidelity PCR system mix (Roche), and 100nM (each) primer were amplified. PCR was performed with the GeneAmp PCR System 9700 (Perkin Elmer, Wellesley, Mass.) with an initial denaturation step of 95°C for 15 min, followed by 40 cycles of 95°C for 15 s and 62°C for 1 min.

### Determination of H_2_O_2_ production

Since is reported that the lack of vaginal H_2_O_2_ producing lactobacilli is associated with bacterial vaginosis, the *Lactobacillus* isolates were also characterised for their production of H_2_O_2_. The capacity of *L. crispatus* L1 to produce H_2_O_2_ was tested with a semiquantitative assay on tetramethylbenzidine agar plates [15] using Brucella agar (Difco) containing 0.001% (w/v) horseradish peroxidase (Sigma), 0.023% (w/v) tetramethylbenzidine (Sigma) and 1% (w/v) starch. This medium was supplemented with 0.5 mg of bovine haemin (Sigma) and 0.1 mg of vitamin K1 (Sigma) in 100 ml of final volume. Serial dilutions of lactobacilli were inoculated in the medium and incubated in anaerobic conditions at 37°C for 72 h. Plates were then exposed to ambient air and H_2_O_2_-producing colonies were revealed by the appearance of a blue colour. According to the colour intensity, the strains were classified as strong, medium, weak or negative (white colonies) producers [41].

### Gastrointestinal survival: simulated gastric and pancreatic juices

Shake flask experiments were performed to evaluate the capability of *L. crispatus* L1 to survive the gastrointestinal tract. Simulated gastric and pancreatic juices were prepared by slightly modifying the protocols reported by Kos and colleagues [42]. Briefly, gastric juices were simulated with a solution of NaCl, 125 mM, KCl 7 mM, NaHCO_3,_ 45 mM and pepsine (Sigma Aldrich) 0.3% (w/v), with a final pH equal to 2 obtained by HCl addition. Either 6.0∙10^8^ cells · ml^−1^ (low dose, minimal starting density for shake flasks experiments necessary to avoid the lag phase) or 1.8·10^9^ cells · ml^−1^ (high dose, typical amount delivered in probiotic commercial products) were inoculated into the medium and incubated 2–3 h in shaker at 37°C and 110 rpm to simulate physiological conditions. This step was followed by centrifugation (15 min at 1200 × g) and re-suspension of the cells in a solution containing pancreatine (Sigma Aldrich) 0.1% (w/v), Oxgall bile (Sigma Aldrich) 0.15% (w/v) with a final pH equal to 4, to simulate pancreatic juices. The suspension was incubated for 3 h, after which cells were centrifuged and re-suspended in fresh MRS medium to evaluate bacterial growth. At the end of each step cell viability was measured by plating aliquotes and counting colony forming units (cfu).

### Fermenter experiments

The fermenter used was a Biostat CT, Braun Biotech International (Melsungen, Germany), 2 l working volume, equipped with a digital control unit and connected to a PC for remote control via MFCS-win software. *L. crispatus* L1 was grown at T = 37°C, pH = 6.5. The stirring velocity was initially set to 100–200 rpm and increased up to 300 rpm during the experiment. The medium was sparged with nitrogen after sterilization prior to inoculation for at least 30 min. Experiments in batch mode were carried out using the SDM medium, controlling the pH by automatic addition of NH_4_OH (2.5 M).

MF experiments were performed using SDM, starting in batch mode, and switching after 8–10 h to fed-batch and approximately 4 h later to MF mode. The duration of each phase was set based on lactate formation, carbon source consumption rate and their influence on growth rates. Filtered exhaust medium was replaced with a fresh salt solution with a level controller, to maintain a constant fermentation volume. Microorganisms were therefore held in the vessel and fed with appropriate profiles generally ranging from 1 to 5 g · l^−1^ · h^−1^. However, differently from previous data [34], the C/N ratio in the nutrient solution was lowered from 1/4 to 1/16 during the MF phase to further decrease the impact of raw materials on process costs.

A Biostat C Braun Biotech International (Melsungen,Germany) bioreactor with a 15 l working volume was used for the production of exopolysaccharides. Two repeated batch experiments were carried out using SDM medium as previously described, in order to purify higher amounts of EPS to allow extensive structural characterization.

### Analytical methods

Cell growth was followed during experiments by measuring absorbance at 600 nm on a Beckman DU 640 Spectrophotometer (Milan, Italy). Samples collected every hour were spinned down in an ALC PK 131R centrifuge at 2000×g, and the wet weight was measured after centrifugation and washing in saline solution (0.9% NaCl w/v). The washed pellet was dried overnight (16–18 h) at 85°C and a calibration curve relating the absorbance value to the cell dry weight was generated. One gram per litre of dry cell weight corresponded to 1.9 OD_600_. This correlation was extrapolated on many different fermentation experiments. Cell number was also measured by direct counts at the optical microscope and plating for viability determination (cfu). The supernatant (1 ml) was ultrafiltered on a centricon tube (10 KDa Mw cut–off, Millipore) at 5000×g to prepare the samples for analytical quantification. The concentration of glucose, or other carbon sources, was measured through HPAEC-PAD analysis performed with a Dionex chromatographer (model DX 500); the organic acids from the culture broth and the permeate solutions were analysed by HPLC as previously described [34]. A quick off-line determination was obtained for glucose by using the Haemo-Glukotest 20–800 stripes (Boehringer-Manheim, *In vitro* diagnosticum).

### EPSs purification and quantification

EPSs were collected and isolated from fermentation supernatants of *L. crispatus* L1. To quantify EPSs during growth, opportunely diafiltered supernatants were assayed using the anthrone/H_2_SO_4_ method [43], using a glucose solution as standard. After harvesting (e.g. 24 h) removal of cells was obtained by centrifugation (2000 × *g* 30 min) and the supernatants were recovered to purify EPSs.

The developed downstream procedure consisted in a pre-treatment of the fermentation supernatant with 4U per litre of protease (*Aspergillus oryzae* 3.2 U⋅mg^−1^, Sigma) for 60 min at room temperature followed by membrane-based UF and DF steps. The procedure was carried on Uniflux-10, (GE Healthcare, USA) an automated tangential flow filtration pilot system also equipped with a sensor level to perform fed-batch concentration or constant volume DF; temperature probe on the retentate line; pressure sensors and flow meters on feed, retentate and permeate lines; pH meter, conductivity meter and UV (with λ = 280 nm) detector on the permeate line. It is connected to a PC and a UNICORN TM software, that allows to control, manage and monitor the process and its parameters. The supernatant was ultrafiltered on 5KDa membranes with a filtering area of 0.1 m^2^ and diafiltered with 5 volumes of distilled water. After addition of 0.08 M NaCl the recovered retentate was precipitated with 6 volumes of acetone and ethanol (1:1 v/v). The precipitate was dried, resuspended in sterile water and treated with active charcoal to decolorization and purification from accidental endotoxin contamination. Finally the concentrated EPS solution was microfiltered on 0.22 μm membranes and lyophilized. The powder obtained was used for further characterization.

### General analytical and spectroscopic methods

Determination of sugars residues and of their absolute configuration, GLC and GLC-MS were all carried out as described. 1D 2D NMR experiments were carried out as described [44,45].

### Culturing of Vk2/E6E7cells

Vk2/E6E7, immortalized human vaginal epithelial cell line (American Type Culture Collection), were grown in 75-cm^2^ flasks (Falcon, Becton Dickinson Biosciences, Milan, Italy) at 37°C (5% CO_2_) in Keratinocyte-Serum Free medium (GIBCO-BRL San Giuliano Milanese, Milan, Italy) with 0.1 ng∙ml^−1^ human recombinant EGF, 0.05 mg∙ml^−1^ bovine pituitary extract, and additional calcium to a final concentration of 0.4 mM. The medium was changed every 2 days. Confluent monolayers (2.5 × 10^5^ cells) were grown in six-well tissue culture plates (Falcon, Becton Dickinson Biosciences, Milan, Italy) in Dulbecco’s modified Eagle’s medium and Ham’s F12 medium (D-MEM) (GIBCO-BRL San Giuliano Milanese, Milan, Italy), antibiotic-free and FCS-free, for 24 h, before starting experiments. One million Vk2/E6E7 cells/well were used for the adhesion assay.

### Adhesion of *L. crispatus* L1 to Vk2/E6E7 cells and competition with *C. albicans* for adherence

Cell suspensions of *L. crispatus* L1 were grown in MRS broth at 37°C in anaerobic conditions.

*C. albicans* was identified on the basis of growth characteristics, colony morphology, cellular appearance, and carbohydrate assimilation patterns using commercially available ATB ID 32 C test kit (bioMérieux, Marcy/Etoile, France) at the Operative Unit of Microbiology, Second University of Naples, Italy. Yeast cells were prepared by inoculating four colonies isolated from Saburaud agar (Oxoid, Milan, Italy) plates in 6 ml Brain Heart infusion broth (BHI broth) (Oxoid, Milan, Italy), and incubating the suspension at 30°C for 18 h under constant shaking. These conditions yield cultures composed primarily of blastospores at the late exponential growth phase.

Cultures of microorganisms were collected by centrifugation from the broth cultures, washed three times and finally suspended in phosphate-buffered saline (PBS; pH 7.1). The working dilution of the microorganism suspensions was determined by performing sequential measurements of optical densities of cultures at 600 nm and quantification of viable microorganisms by colony counts. For each strain, the correlation between the OD_600_ and cfu was established. The microorganism cells suspended in DMEM were used for the adhesion and interference assays.

Adherence of *L. crispatus* L1 to Vk2/E6E7 cells was assayed by a method described previously with slight modifications [46]. Preliminary experiments using 10:1, 100:1, and 1000:1 multiplicities of infection (MOI) were conducted to determine the optimal bacterial-to-epithelial cell ratio in our adhesion model. These pilot investigations demonstrated a saturation of adhesion of *L. crispatus* L1 to Vk2/E6E7 cells at a MOI of 10:1. Therefore, for all subsequent adhesion experiments described in this study a MOI of 10:1 was utilized.

Interference experiments were performed with *C. albicans,* a potential vaginal pathogen, that showed a significant capacity to adhere to host cells. The procedures described by Osset et al. [47] were used, with some modifications. For exclusion tests, 1×10^7^ lactobacilli and vaginal epithelial cells were incubated together for 1 h at 37°C in microaerophilic conditions; afterwards, *C. albicans* cells were added, and incubation was further continued for 1 h. During competition tests, 1×10^7^ lactobacilli and 1×10^7^*C. albicans* were mixed and Vk2/E6E7 cell monolayers then inoculated and incubated for 1 h at 37°C in microaerophilic conditions. For displacement tests, 1×10^7^*C. albicans* and epithelial cells were incubated together for 1 h at 37°C in microaerophilic conditions. Successively, 1×10^7^ lactobacilli were added and incubation was prolonged for 1 h. Vk2/E6E7 cells were scored for the presence and number of bacteria and *C. albicans* attached, and cell observation was performed as indicated above.

For exopolysaccharide**-**interference experiments, Vk2/E6E7 cell monolayers were treated with EPS as follows: for competition tests, exopolysaccharide (0.01-0.1-1.0 mg∙ml^−1^) and 1×10^7^*C. albicans* were mixed and, successively, Vk2/E6E7 cell monolayers were inoculated and incubated for 1 h at 37°C in microaerophilic conditions. For exclusion tests, vaginal epithelial cells were pre-treated with EPS (0.01-0.1-1.0 mg∙ml^−1^), before addition of the *C. albicans* suspension for 1 h at 37°C in microaerophilic conditions. At the concentrations used, the EPS did not affect epithelial cell viability. In preliminary experiments monolayers were pre-treated with EPS for 1, 4, 6 and 18 h at 37°C in microaerophilic conditions.

Microorganism adhesion to Vk2/E6E7 cells was assessed by microscopy (×100) after Gram’s stain by counting the number of micro-organisms attached to 30 consecutive cells. The results of the three conditions (i.e. exclusion, competition and displacement) were expressed as the average number of *C. albicans* per Vk2/E6E7 cells and compared with adhesion without lactobacilli or EPS (control value). The control values were taken as 100% of adhesion and the inhibition of *C. albicans* adherence was calculated by subtracting each adhesion percentage from its corresponding control value.

Adhesion experiments were conducted three times with at least three replicates per group. A difference in mean values was deemed significant if the P values were <0.05 or highly significant if the P values were <0.01. The three experimental groups were compared using a one-way analysis of variance. Post hoc group comparisons were conducted using the Student-Newman-Keuls test.

### HBD- 2 ELISA

Semi-confluent Vk2/E6E7 were grown in six-well tissue culture plates and were treated with EPS (0.01-0.1-1.0 -5.0 mg∙ml^−1^) for 18 h. Cell-free supernatants were recovered by centrifugation and assayed to establish the concentration of Human beta-defensin 2 (HBD-2) by an enzyme-linked immunosorbent assay (Phoenix Pharmaceuticals, Inc.).

The data were presented as means ± standard errors. All pair wise comparisons were examined using unpaired Student’s two-tailed t-test. Differences were considered significant when P ≤ 0.05.

## Competing interests

The authors declare that they have no competing interests.

## Authors’ contributions

GD, CS and MDR conceived the study. DC, GD and CS drafted the manuscript. GD, AM, DC CDC, VV and VDG performed experiments. All authors read and approved the manuscript.
